# Ultrastructural Analysis of a Forming Embryonic Embodiment in the Adult Zebrafish Optic Tectum Surviving in Organotypic Culture

**DOI:** 10.3390/neurosci3020014

**Published:** 2022-04-02

**Authors:** Ricardo L. Peguero, Nicole A. Bell, Andras Bimbo-Szuhai, Kevin D. Roach, Zoltan L. Fulop, Christopher P. Corbo

**Affiliations:** Laboratory of Developmental Brain Research & Neuroplasticity, Department of Biological Sciences, Wagner College, Staten Island, NY 10301, USA; ricardo.peguero@wagner.edu (R.L.P.); nicole.bell@wagner.edu (N.A.B.); andras.bimbo-szuhai@wagner.edu (A.B.-S.); kevin.roach@wagner.edu (K.D.R.); zoltan.fulop@wagner.edu (Z.L.F.)

**Keywords:** zebrafish, plasticity, brain development, optic tectum, brain regeneration

## Abstract

It has been shown that adult zebrafish are capable of regenerating regions of the central nervous system (CNS) after insult. Unlike in higher-order vertebrates where damage to the CNS leads to glial scar formation and permanent functional deficits, damage to the adult zebrafish CNS is transient and followed by nearly complete reconstitution of both function and anatomy. Our lab’s previous work has shown that explants of zebrafish optic tectum can survive in organotypic culture for up to 7 days, and that at 96 h in culture, regenerating cells of the tectum begin to form structures that resemble the embryonic neural tube seen in vertebrate development. The current project aims to elucidate the cellular and ultrastructural components of the formation of this neural tube-like structure using scanning and transmission electron microscopy. Our results show that after injury and cultivation for 96 h, the explants contained differentiating cells that were undergoing several cellular events, such as neovascularization, and rosette/cisternae formation, leading to the formation of a structure resembling the embryonic neural tube. Additionally, we demonstrate healthy cellular ultrastructures in both degenerated and regenerated areas of the explant.

## 1. Introduction

In the last several decades, zebrafish (Danio rerio) have become a popular laboratory animal for developmental biology, specifically brain development. Zebrafish are a unique vertebrate model organism; they are small, easy to care for, inexpensive, and space efficient. For these reasons, they have been adopted for many areas of research and the use of adult zebrafish has become more prominent [1,2,3,4,5,6,7,8].

Since zebrafish are low on the vertebrate phylogenetic tree, they possess higher regenerative capacity than the more advanced orders [2,3,5,6,9,10]. Previous studies have shown that different regions of the zebrafish central nervous system demonstrate incredibly robust regenerative processes that result in the full resolution of any tissue damage sustained from injury [10,11,12,13]. Such regenerative processes are not seen in mammals and the neurogenic capacity of the mammalian brain has also been shown to be lesser than that of zebrafish [14]; while the zebrafish brain contains several areas of neurogenic activity that are present during adulthood, the adult mammalian brain’s neurogenic activity is restricted to the hippocampus and olfactory bulb [14,15]. Additionally, the zebrafish CNS provides an environment that is supportive of newly born neurons in adulthood whereas the mammalian CNS does not [14,15]. Presumably, the lack of reactive gliosis and glial scar formation make the zebrafish CNS more permissive to regeneration after injury [10,14].

Neurotrophic factors, such as brain-derived neurotrophic factor (BDNF) and nerve growth factor, have been shown to be upregulated in several areas of the zebrafish CNS after injury and during development [16]. In addition to this, several molecular mechanisms and cellular components implicated in the zebrafish neuroregenerative process have been interrogated; primarily, the roles of signaling pathways, including the IL-6/Stat 3, and Notch pathways [17,18], as well as the role of glial and neural progenitor cells have been described [12]. This work suggests that signaling pathways and molecules related to embryonic development mediate the neuroregenerative response on a molecular level while radial glia and neural progenitor cells are responsible for repopulating and reconstructing any insulted areas. 

A study by Tomizawa and colleagues demonstrated that a whole zebrafish brain was able to survive in organotypic culture for seven days [9]. In another study, Kustermann and colleagues showed that neurons from explants of zebrafish retinas were able to survive and regenerate in organotypic culture [19]. Our lab has long been focused on the zebrafish brain cellular anatomy, in particular the structure of the optic tectum [20]. The work by Tomizawa and colleagues as well as Kustermann and colleagues spurred an interest in our group to analyze how pieces of the optic tectum could survive in organotypic culture. The act of removing the pieces would be a model of brain injury in itself.

Our lab showed that zebrafish optic tectum can survive in organotypic culture for up to seven days, even when cut into four separate pieces [3]. While many cells died early in the culture, there were many that survived. After four days in organotypic culture, surviving cells were able to be recruited and form structures resembling the early development of the embryonic neural tube/fold. These forming structures will herein be referred to as embryoid embodiments.

Our initial analysis focused on the cellular events that occurred over the seven days in culture as well as the cellular structure of the embryoid embodiments. This work set out to analyze this time course utilizing transmission and scanning electron microscopy. We investigated regions that appeared to be degenerating as well as the forming embryonic embodiments. We looked into cellular ultrastructure in dying, surviving, and regenerating regions of the cultured tissue pieces.

## 2. Material and Methods

### 2.1. Animal Care and Utilization

This project adhered to the guidelines set forth in the Guide for Care and Use of Laboratory Animals, 8th edition as well as euthanasia protocols in The Zebrafish Book, 5th edition. All zebrafish were obtained from a local pet store in Staten Island, NY and were maintained in a 50-gallon aquarium with a regular day/night cycle (14L:10D) and proper aeration and filtration. Zebrafish were fed once per day with dry tetra min flake food for tropical freshwater fish.

This study utilized 12 adult zebrafish of mixed sex. Each fish generated four explant tissue samples, totaling 48. The explants of three animals (totaling 12 cultured pieces) were processed for scanning electron microscopy (SEM) and those of the other nine animals (totaling 36 cultured pieces) were processed for transmission electron microscopy (TEM). SEM samples were fixed at 24, 48, and 96 h of cultivation. All TEM samples were fixed at 96 h of cultivation.

### 2.2. Culture Media and Surgical Procedure

All surgical techniques were carried out using aseptic conditions. All fish were anesthetized before surgery using a 0.04% tricaine solution. Complete unconsciousness was determined using forceps to pinch the tailfin.

According to our previously published protocol, the brains of the fish were removed, and the tectum was cut into four pieces [3]. Briefly, after anesthesia, the fish were secured, and the skull was removed followed by complete brain extraction. The brain was transferred to organotypic culture media where the optic tectum was cut into four pieces, transferred to Millipore organotypic culture insert (Millipore Sigma, Burlington, MA, USA, cat# PICM03050), and cultivated in the same organotypic culture medium with 5% CO2 at 37 °C.

The organotypic media recipe, adopted from Tomizawa et al., was made up of minimal essential media (MEM) supplemented with 15% horse serum, 15% Hank’s Balanced Salt Solution, 0.2 mM L-glutamine, 50 mg/mL of glucose, and 100 units of penicillin/streptomycin for bacterial inhibition (all ingredients for culture media are from Fisher Scientific, Waltham, MA, USA) [9].

### 2.3. Scanning Electron Microscopy Histotechniques

All tissue explants were fixed in Karnovky’s fixative [21] for at least 24 h, post-fixed in 1% osmium tetroxide (Fisher Scientific, Waltham, MA, USA) for two hours, dehydrated through an increasing ethanol series, and further dried using two treatments with propylene oxide followed by complete desiccation with hexamethyldisilazane (HMDS) (Fisher Scientific, Waltham, MA, USA). The processing vial caps were removed and replaced with aluminum foil and one hole was punctured into foil to allow for slow evaporation of the HMDS in the fume hood for at least 24 h. 

Once completely dry, the samples were mounted on aluminum SEM stubs using sticky carbon adhesives. The mounted samples were coated using a Hummer IV (LADD research, Williston, VT, USA) with the sputter coater set at 100 mT of vacuum and 10–15 mA for 10 min. Samples were imaged in a Topcon ABT- 32 SEM (Topcon, Livermore, CA, USA) equipped with an Orion digital imaging system (Topcon, Livermore, CA, USA).

### 2.4. Transmission Electron Microscopy Histotechniques

All tissue explants were fixed in Karnovsky’s fixative (4% paraformaldehyde, 2% glutaraldehyde, pH 7.2) for at least 24 h, post-fixed in 1% osmium tetroxide for two hours, dehydrated through an increasing ethanol series, embedded in Durcupan resin (Millipore Sigma, Burlington, MA, USA,), and polymerized overnight at 60 °C.

Blocks were trimmed by hand, and 1 µm light sections were collected using a Reichert OMU-2 ultramicrotome (Reichert, Depew, NY, USA) and glass knives. Sections were stained with 1% toluidine blue and observed using an Olympus BX40 light microscope (Olympus scientific solutions, Waltham, MA, USA). Ultrathin sections were collected using a Sorvall MT-6000 ultramicrotome (Sorvall, Waltham, MA, USA) and a diamond knife. Silver/gold sections were spread with xylene and collected on 2 mm copper slot grids coated with 0.5% formvar. Grids were contrasted with 2% uranyl acetate and 0.1% lead citrate and carefully washed so as not to disturb the formvar coating. Grids were dried prior to imaging on a Philips CM100 TEM (Mount Holyoke, South Hadley, MA, USA) equipped with a Gatan Orius digital imaging system (Gatan, Pleasanton, CA, USA). Montages were assembled in Adobe Photoshop.

## 3. Result

In this paper, we present an ultrastructural analysis of zebrafish optic tectum maintained in organotypic culture. Scanning electron microscopy was used to analyze surface structures at all time points, while the transmission electron microscopy ultrastructure was focused on samples in culture for 96 h. This time point was selected based on our previous work where we demonstrated that the earliest time when the forming embryoid embodiments could be detected was 96 h. Scanning electron microscopy was used to characterize the surface structure, analyze cells that had migrated to the periphery, and to determine if we could detect any newly forming structure on the surface of the tissue piece.

**Figure 1:**
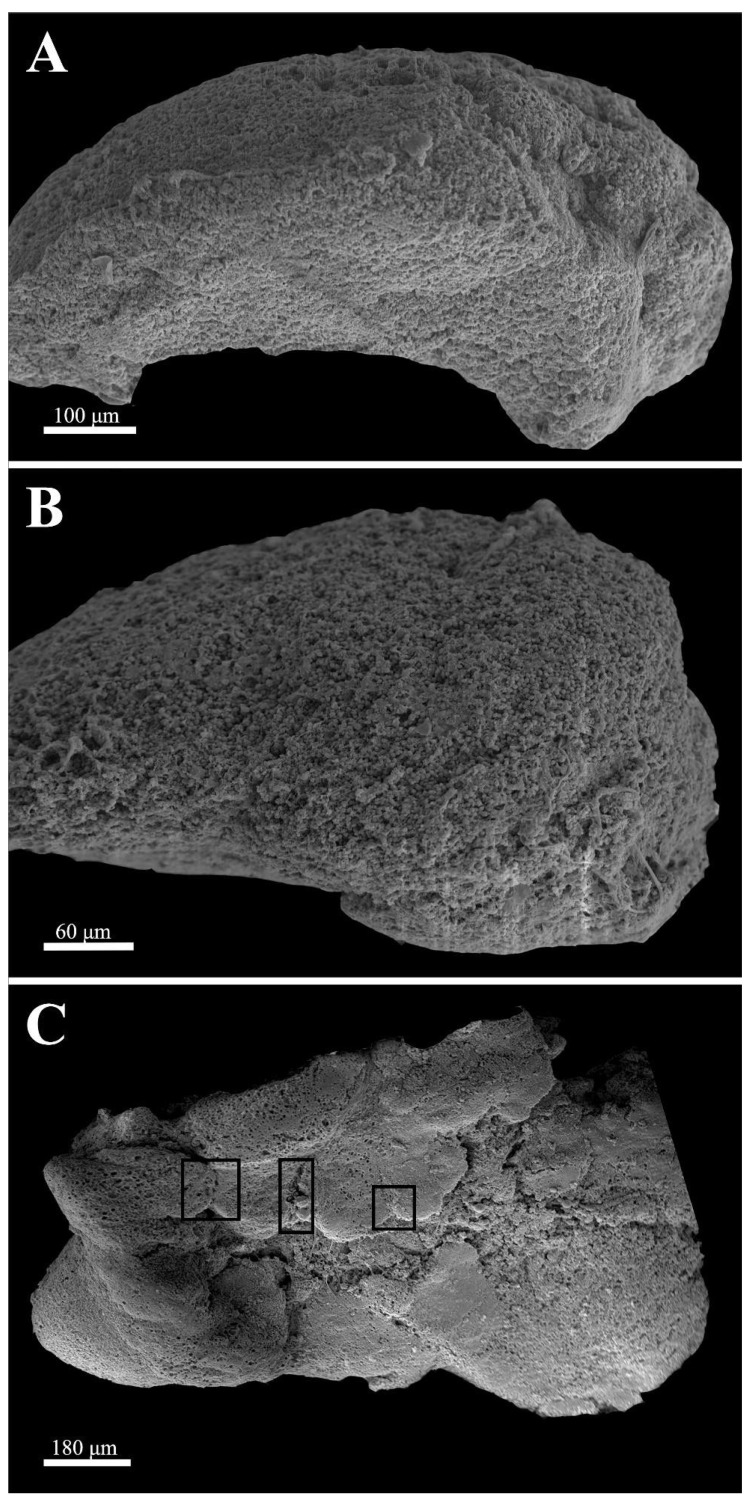
Scanning electron micrographs of three surviving samples representing three time points in culture. At 24 h (**A**), all structural characteristics of the zebrafish optic tectum could be recognized. At 48 h (**B**), only the ependymal layer was recognizable as a distinct morphological entity but the whole surface tectal piece was covered with densely packed round cells. At 96 h (**C**), symmetrical protrusions of regenerating tissue formed on the surface of the explant. These structures usually have a midline, a raphe-like groove (black boxes) that divides the formations into two mirrored halves.

Figure 1 depicts scanning electron micrographs of surviving samples at 24, 48, and 96 h, respectively. At 24 h, the ependymal layer, the subventricular zone, and the rest of the cortical region [20] up to the pial surface could be recognized and no general anatomical distortion could be detected. At 48 h in culture, only the ependymal layer was recognizable as a distinct morphological entity. The surface tectal piece was covered with densely packed round cells, which were hard to differentiate. However, after 96 h in culture, different, symmetrical protrusions appeared to have formed on the surface. These structures usually have a midline, a raphe-like groove that divides the formations into two mirrored halves. In the presented sample, three of these formations were formed (boxes). We should note that in our many samples, the number of these formations varied greatly from none to several per piece. The formations in these samples represent the surface of the forming embryoid embodiments.

**Figure 2:**
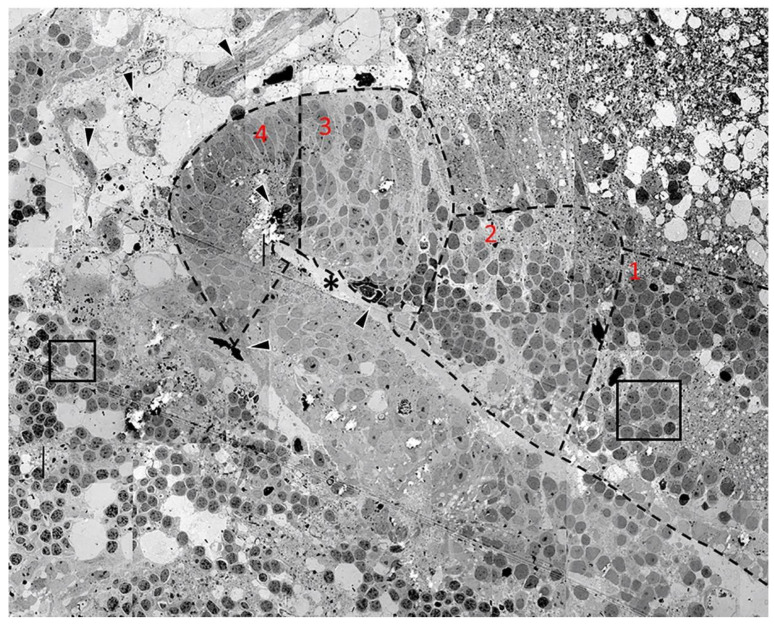
Electron microscopic panoramic overview of a folding embryoid embodiment in adult zebrafish optic tectum surviving in organotypic culture for 96 h. The image is a montage of 50 electron micrographs. Areas 1–4 represent cells differentiated to varying degrees, with area 4 representing the most highly differentiated cells. The central, organized region (asterisk) of the image, which is surrounded with spongiform degenerative regions (upper right corner) intermingled with signs of neovascularization and hematopoiesis (arrowheads) as well as rosettes (black boxes), represents early developmental formations. Inferior to the ventricular space, some mitotic cells can be recognized among differentiating nuclei.

Figure 2 is a panoramic overview of a folding embryoid embodiment in adult zebrafish optic tectum surviving in organotypic culture for 96 h. The image is a montage of 50 electron micrographs captured at approximately 3000× magnification. While the 96-h samples contained larger numbers of advanced formations, they also contained several earlier stages of cell grouping, differentiation, and migration, making it a good time point for detailed morphological analysis. Figure 2 depicts an advanced embryoid embodiment formation in which a forming “ventricular space” can be found. This formation is surrounded by many undifferentiated cellular arrangements and samples of neovascularization.

The embryoid embodiment in the middle of Figure 2 is sectioned in the longitudinal plane and the forming ventricular space is visible at the center of the structure (asterisk). A forming ependymal layer could be seen lining the “ventricular space” and microvilli could be detected; these are shown in greater detail in a subsequent figure. Cells in the forming embryoid embodiment showed an increasing level of differentiation toward the tip of the structure. We labeled each level of differentiation with a number (1–4), with 4 being the most differentiated.

The most advanced tissue, found in region 4, contained both advanced ependymal cells and signs of neovascularization (arrowheads) in which newly formed blood cells could also be detected.

Cells in region 3 clearly resembled pseudostratified columnar neuroepithelium typical for the normal development of the healthy vertebrate embryonic brain. The nuclei of these cells could frequently be detected as one elongated cytoplasm that was interconnecting the ependyma and the forming pia mater. This was indicative of the structure and role of the radial glial cells in normal vertebrate brain development. While the pia mater of the forming cortical structure [20] above the ventricular space was clearly recognizable, the pia mater of the forming region below the ventricular space was hardly recognizable. Immediately to the right of this region, region 2 could be seen. This group of cells represents an earlier stage of neuroepithelial formation but, at this position, we did not see a formed pial surface; rather, the cells interacted with a region of spongiform degeneration. The nuclei of these cells were more rounded and several of them were in a stage of cell division. Mitotic figures could still be recognized in several cells.

In region 1, cells forming rosette groupings of rounded cells with dark nuclei could be detected within close proximity to spongiform degeneration. These rosettes are the earliest significant formation leading to the start of brain development in vertebrates [22,23]. These cells, under some chemical influence, group into rosette formations and seem to subsequently form more advanced developing cortical structures [20]. The region of the embryoid embodiment that was inferior to the developing ventricle was not developed, as was the case for its previously described superior counterpart. However, this region did display homogeneous lightly colored nuclei as well as several mitotic cells.

At the upper right corner of the image, an area of spongiform degenerating tissue could be seen with numerous white spots which were locations of former neurons. However, in that region, several small cells with small, dense, dark nuclei could be seen. Among these surviving elements, groups of cells could be seen forming classical rosette arrangements around clear cisternal spaces [3,20]. The nuclei of the cells forming rosettes had different arrangements of chromatin, which is a way to distinguish different cells. Most of the nuclei had a homogenous chromatin with one or two nucleoli. Some of the other nuclei had a distorted shape and dark appearance with recognizable hetero and euchromatin arrangements, similar to the nuclei in other rosettes recognized below the neural fold formation. The left sides of the images displayed several distinct populations of cells grouped together.

Cells in small rosettes could be seen in the lower region of the image (box). These cells had varying densities and were not homogenous, displaying clearly visible hetero and euchromatin. These cells formed the rosette around a cisternal space within the tissue. In the upper left corner, it was possible to see a grouping of cells that were larger than any of the previously mentioned small rosettes. These cells also had varying nuclei colorations, and some cells had nucleoli. Cisternal spaces could be seen at either end of the structure. We hypothesize that this formation was a more advanced form of the simple rosettes below them.

Lastly, there were several events of neovascularization detected both within the embryoid embodiment as well as in the surrounding areas (arrowheads). Some of these vessels were in a more advanced stage of development and, in some cases, showed the presence of newly formed blood cells.

**Figure 3:**
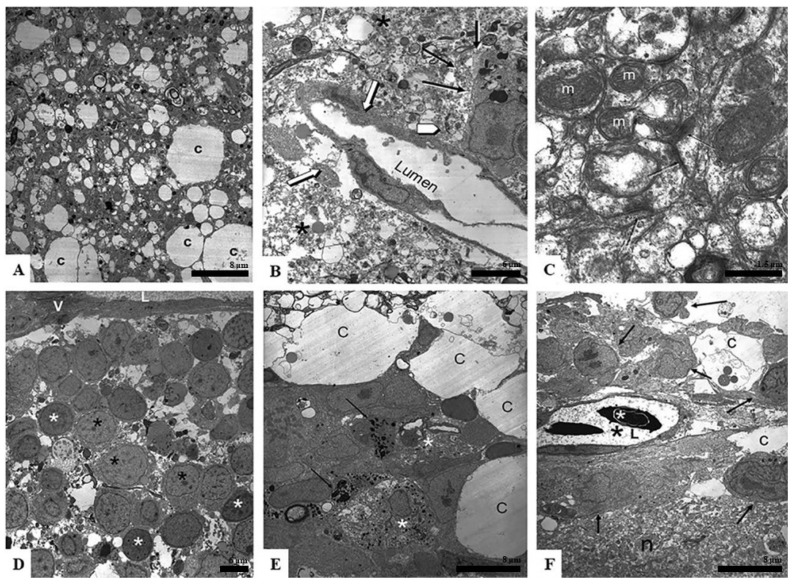
Ultrastructural characteristics of regenerating optic tectum after 96 h in culture. Specifically, (**A**) presents a typical sample of spongiform degenerating tissue with a large number of varying size white spaces, as well as larger cisternal spaces (c). (**B**) shows a surviving blood vessel absent of any blood cells (white arrows). The surrounding area was greatly degenerated (asterisks). The black arrows point to a surviving cell in the area. The white arrowhead points to dense granules. (**C**) shows an area of degrading neuropil. Healthy mitochondria (m) with intact cristae and healthy synapses (black arrows) can be seen. (**D**) is an area of surviving cells that grouped together in a region close to a large vessel seen at the top of the image (V and L). The cells display nuclei of different densities (black asterisk—lighter nuclei; white asterisk—darker nuclei). (**E**) shows an area of early regeneration. Mast cells could be detected (black arrows—granules; asterisk—mast cell body) in this area. The mast cells were surrounded by healthy- appearing cells and cisternae (c). (**F**) shows an area of neuropil (n) that appears more intact than those seen in (**B**,**C**). One can also see a healthy blood vessel in which new blood cells have formed (white asterisk—red blood cell nuclei; black asterisk—fluid portion of blood vessel; L—lumen of blood vessel). The black arrows are pointing at cells that were active around the vessel.

**Figure 4:**
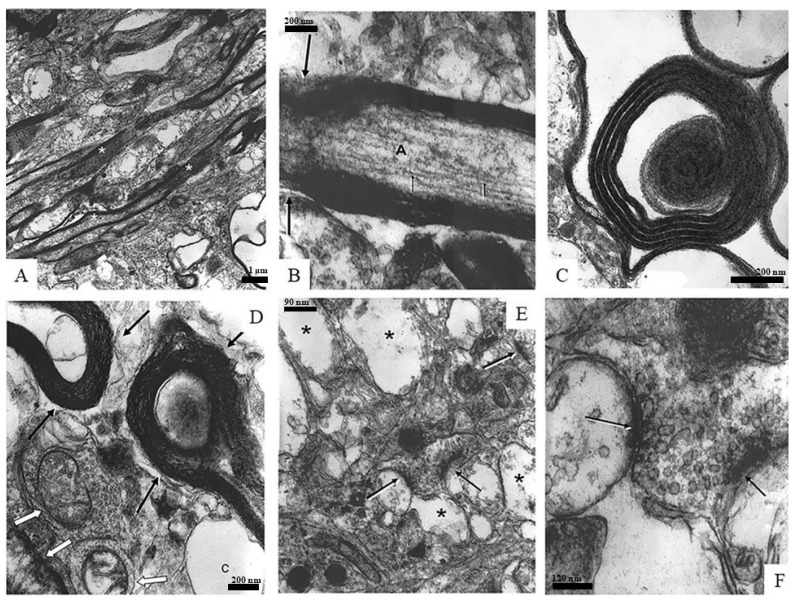
Panel of images depicting different samples of neuronal components found in the spongiform neuropil, such as myelinated axons, synapses, and mitochondria. (**A**) shows healthy, myelinated axons (asterisk). (**B**) is a high-magnification image of (**A**), in which axoplasm (A) and axonal neurofilaments (arrows) can be easily recognized. (**C**) shows an image where both the axon and tissue surrounding the myelin sheath are deteriorating or are completely missing, while the myelin seems to be intact. The myelin in (**D**) also displays a healthy appearance while the axoplasm of the axons (black arrows) is nearly completely degraded. Cisternae (c) as well as mitochondria (white arrows) can also be seen. (**E**,**F**) focus on surviving synaptic elements (arrows). (**E**) depicts these synaptic elements among degenerative tissue (asterisks). (**F**) is an enlargement of the two synapses seen in the center of (**E**).

**Figure 5:**
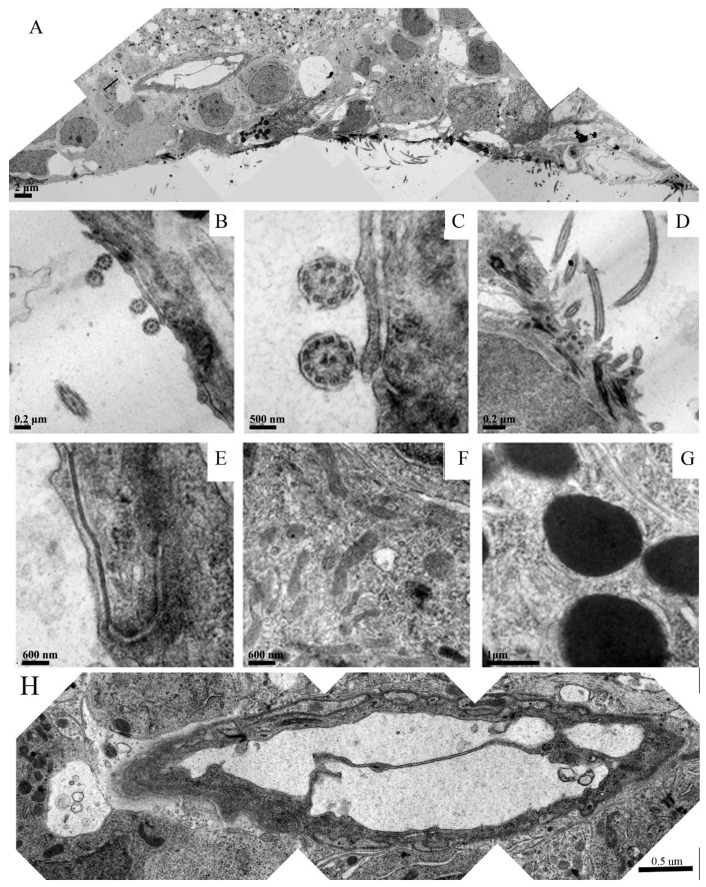
The different regions of the forming ependymal layer in the embryoid embodiment. (**A**) is a montage showing newly formed ependymal cells with their microvilli protruding into the ventricular space. Neovascularization can also be recognized. (**B**–**D**) depict longitudinal and cross sections of the ependymal microvilli within the neural groove. (**E**) depicts a well-formed tight junction between two neighboring ependymal cells. (**F**) shows that the cytoplasm of these ependymal cells were loaded with healthy mitochondria and other organelles. Some of the forming ependymal cells that were imaged were loaded with dark inclusions, which are shown in (**G**). (**H**) is a montage of the venous blood vessel seen in (**A**) at a higher magnification.

Figure 3, Figure 4 and Figure 5 present ultrastructural samples from different regions of several tissue blocks representing dying, surviving, and newly formed elements seen after 96 h in organotypic culture. Figure 3A presents a typical sample of spongiform degenerating tissue with a large number of varying size white spaces, likely the location of former cells now dead or migrated away. These spaces were closely associated with small dark dots that were the remnants of former cells. The larger areas were cisternal spaces (c), which were likely assisting in nutrient movement through the tissue piece.

Figure 3B shows a surviving blood vessel absent of any blood cells (white arrows). One can see that the surrounding area was greatly degenerated (asterisks) and the internal lining of the vessel was blebbing off into the luminal space, likely because it was degrading. The black arrows point to a surviving cell in the area. This was likely a macrophage because of its proximity to the blood vessel and the large amount of internal cellular inclusions formed as the cell phagocytized the dying regions. The white arrowhead points to dense granules, likely cellular remnants of apoptosis.

Figure 3C shows an area of degrading neuropil. Interestingly, even in areas of spongiform degeneration, one can find healthy mitochondria (m) with intact cristae and the presence of healthy synapses (black arrows).

Figure 3D is an area of surviving cells that grouped together in a region close to a large vessel seen at the top of the image (V and L). The cells displayed nuclei of different densities (black asterisk—lighter nuclei; white asterisk—darker nuclei). 

Figure 3E shows an area of early regeneration. One can detect mast cells (black arrows—granules; asterisk—mast cell body) in this area. The mast cells were surrounded by healthy-appearing cells, one of which was in an active phase of mitosis. As seen in Figure 2, these cells were grouping around large cisternal spaces (c).

Figure 3F shows an area of neuropil (n) that appears to be more intact than those seen in 3B and C. One can also see a healthy blood vessel in which new blood cells formed (white asterisk—red blood cell nuclei; black asterisk—fluid portion of blood vessel; L—lumen of blood vessel). The black arrows are pointing at cells that were metabolically active around the vessel.

Figure 4 is a panel of images depicting different samples of neuronal components found in the spongiform neuropil, such as myelinated axons, synapses, and mitochondria. Figure 4A,B show myelinated axons (asterisks) in longitudinal sections: A is a low-magnification overview and B is a high-magnification image in which axoplasm (A) and axonal neurofilaments (arrows) can be easily recognized. Figure 4C,D depict cross sections of myelinated axons. In Figure 4C, both the axon and tissue surrounding the myelin sheath are deteriorating or are completely missing, while the myelin seems to be intact. The myelin in Figure 4D also displays a healthy appearance while the axoplasm of the axons (black arrows) is nearly completely degraded. It is interesting to note that next to these axons, surviving mitochondria can be seen (white arrows). Figure 4E,F focus on synaptic elements (arrows): E is a low-magnification overview of the region in a spongiform degenerating neuropil (asterisks) while F is an enlargement of the two synapses seen in the center image E. The axon terminals seen in Figure 4F have well-recognizable cell membranes and are loaded with healthy-appearing synaptic vesicles that clearly contain neurotransmitters.

Figure 5 is a panel that focuses on different regions of the forming ependymal layer in the embryoid embodiment. Figure 5A is a montage overviewing a region that clearly shows the specific arrangement of the newly formed ependymal cells with their microvilli protruding into the ventricular space. Signs of neovascularization can also be recognized. Figure 5B–D depict longitudinal and cross sections of the ependymal microvilli within the neural groove. Figure 5E depicts a well-formed tight junction between two neighboring ependymal cells. Figure 5F shows that the cytoplasm of these cells were loaded with healthy mitochondria and other organelles determined through their intact membrane structures. Many of the forming ependymal cells were loaded with dark inclusions, which are shown in a higher magnification in Figure 5G. These inclusions were likely glycogen granules. Figure 5H is a montage of the venous blood vessel seen in 5A at a higher resolution. This image demonstrates healthy endothelial cells and the formation of venous valves. The vessel was surrounded by healthy tissue components that might be either smooth muscle or pericytes.

## 4. Discussion

The use of organotypic culture of brain tissue is both a powerful and capricious approach to study the plasticity of traumatized mature zebrafish brains. Taking out the brain from the skull, cutting it up into pieces, and placing it into a foreign environment for organotypic culture is a drastic traumatic brain injury (TBI). Each sample provides a unique view of the regenerating embryoid embodiments depending on the orientation in which they were sectioned. Additionally, it is exciting to see such robust regeneration in a brain tissue explant that had been completely removed from the organism, whereby it was removed from continual blood flow and hormone-like signaling molecules. The entirety of the regenerative process occurred with molecules that were present within the tissue sample itself. There were no additives in the growth media to promote regeneration.

Most work conducted in zebrafish brain regeneration is performed in brain injury models using the telencephalon, retina, or forebrain [24,25]. Our model is distinct in two ways: one, our model focuses on the optic tectum, which is the only cortical structure in the zebrafish brain [20], and second, our model uses the surgical procedure of explant generation as a method to introduce brain injury. Additionally, by allowing our explants to survive ex vivo in culture, any influence from factors outside of the tectum on regeneration are removed.

Upon imaging of the external morphology of the tissue explants using SEM, healthy, surviving tissue could be seen up to 24 h in culture, whereas after 48 h in culture, widespread cell death and spongiform degeneration were most prominent. At 96 h in culture, cellular migration and organization could be detected and regenerating, highly organized embryoid embodiments could be seen forming along a raphe. Cells exhibiting migratory characteristics presumably moved from areas within the explant to the surface to access the nutrient-rich media. Additional factors such as BDNF signaling may also have been implicated in the organized migration observed in our explants. These migrating cells also formed structures that resemble the neural tube/fold as it is seen in normal early vertebrate CNS development [26,27].

When analyzing the ultrastructure of tissue maintained in culture for 96 h, several facets of vertebrate neurulation were clearly recognizable, with the most apparent being the formation of neural tube-like structures. However, as we previously showed in our light microscopic analysis, there were other, smaller events that were evident. Examples of these are rosette formation and signs of neovascularization.

The embryoid embodiment was seen in cross section and a ventricular space could be seen surrounded by an ependymal surface made up of cells, some of which appeared mitotic and differentiated to varying extents; presumably, cells in the explant divided, populated, and organized using the spongiform degenerating areas as a scaffold and then differentiated to form embryonic structures that resembled the vertebrate neural tube. Several levels of differentiation could be detected in these explants. Cells forming rosettes around cisternal spaces were among the least differentiated [28], while more differentiated ependymal cells could be seen directly lining the ventricular space and forming an ependymal layer. Within the new parenchyma, more differentiated cells could be seen forming highly organized groupings, and many of these cells exhibited radial glia-like projections. Neovascularization and pial layer formation were also prominent in the tissue explants cultured for 96 h. Even in areas of spongiform degeneration, both healthy and aberrated synapses as well as axons could be seen among other surviving subcellular elements such as mitochondria. Collectively, our results show that the cellular reorganization of the zebrafish optic tectum after TBI is remarkably similar to the organization seen during vertebrate neural development.

The current literature related to the cellular components of the regenerative process in the zebrafish optic tectum is scarce and even in the more heavily interrogated brain regions such as the telencephalon, little work is focused on analyzing the regenerative process from an ultrastructural perspective; our project provides insight into the regenerative process of the zebrafish optic tectum from a perspective that has received little to no attention. Although our methods were sufficient to describe the ultrastructural characteristics of cell death, neovascularization, cellular organization, and regenerative neurogenesis in the optic tectum after injury, several questions arise from our results, with the most pressing of these questions pertaining to the role of mast cells in the regenerative response. We demonstrated in our previous study that the cells that characteristically appear as mast cells are in fact mast cells due to their metachromasia with toluidine blue staining [3]. Additionally, we characterized the presence of the mast cells at different time points in a separate work [29].

Although we showed that mast cells are present in regenerating tissue explants and are seen associating with other rosette-forming cells and degenerative tissue, little can be said about their function. Since mast cells have been shown to degranulate and, in some cases, mediate regenerative tissue responses including angiogenesis and epithelial tissue repair [30,31,32], our group hypothesizes that mast cells act in a fashion that can both induce and/or mediate the neuroregenerative process through their degranulation. This hypothesis is in part supported by previous work that showed that mast cell activation can induce BDNF expression in microglial cells [33]. Further support for this hypothesis is seen through previous experiments, which demonstrated that several distinct neuropeptides, including nerve growth factor, can both activate and be released by mast cells [34]. Additionally, it was shown that sterile inflammation is sufficient for the initiation of the regenerative response in zebrafish [35]. Because mast cells, through their degranulation, play a role in inflammatory responses, we reason that there may be a connection between mast cell degranulation and inflammatory processes that are necessary in the initiation of the regenerative response in zebrafish. 

In future studies, our group will seek to describe the molecular components of mast cell involvement in neuroregeneration after time in culture. Future work may also concern itself with the characterization of the molecular components of the neovascularization and rosette formation seen in our results to determine the role of newly forming circulatory elements and rosettes. Our group’s work suggests that particular attention should be paid to the processes of early vertebrate neurulation when interrogating the regenerative response in zebrafish after TBI.

## Data Availability

The authors confirm that the data supporting the findings of this study are available within the article.
